# Ginsenoside Rg1 Decreases Aβ_1–42_ Level by Upregulating PPARγ and IDE Expression in the Hippocampus of a Rat Model of Alzheimer's Disease

**DOI:** 10.1371/journal.pone.0059155

**Published:** 2013-03-08

**Authors:** QianKun Quan, Jue Wang, Xi Li, Yi Wang

**Affiliations:** 1 The Key Laboratory of Biomedical Information Engineering of Ministry of Education, and Institute of Biomedical Engineering, School of Life Science and Technology, Xi'an Jiaotong University, National Engineering Research Center of Health Care and Medical Devices, Xi'an Jiaotong University Branch, Xi'an, China; 2 Department of Geriatrics, The Second Affiliated Hospital, Xi'an Jiaotong University College of Medicine, Xi'an, China; Mayo Clinic College of Medicine, United States of America

## Abstract

**Background and Purpose:**

The present study was designed to examine the effects of ginsenoside Rg1 on expression of peroxisome proliferator-activated receptor γ (PPARγ) and insulin-degrading enzyme (IDE) in the hippocampus of rat model of Alzheimer's disease (AD) to determine how ginsenoside Rg1 (Rg1) decreases Aβ levels in AD.

**Experimental Approach:**

Experimental AD was induced in rats by a bilateral injection of 10 µg soluble beta-amyloid peptide 1–42 (Aβ_1–42_) into the CA1 region of the hippocampus, and the rats were treated with Rg1 (10 mg·kg^−1^, intraperitoneally) for 28 days. The Morris water maze was used to test spatial learning and memory performance. Hematoxylin-eosin staining was performed to analyze the hippocampal histopathological damage. Immunohistochemistry, western blotting, and real-time PCR were used to detect Aβ_1–42_, PPARγ, and insulin-degrading enzyme (IDE) expression in the hippocampus.

**Key Results:**

Injection of soluble Aβ_1–42_ into the hippocampus led to significant dysfunction of learning and memory, hippocampal histopathological abnormalities and increased Aβ_1–42_ levels in the hippocampus. Rg1 treatment significantly improved learning and memory function, attenuated hippocampal histopathological abnormalities, reduced Aβ_1–42_ levels and increased PPARγ and IDE expression in the hippocampus; these effects of Rg1 could be effectively inhibited by GW9662, a PPARγ antagonist.

**Conclusions and Implications:**

Given that PPARγ can upregulate IDE expression and IDE can degrade Aβ_1–42_, these results indicate that Rg1 can increase IDE expression in the hippocampus by upregulating PPARγ, leading to decreased Aβ levels, attenuated hippocampal histopathological abnormalities and improved learning and memory in a rat model of AD.

## Introduction

Excessive accumulation of beta-amyloid peptide (Aβ) in the brain is the key pathological change in Alzheimer's disease (AD) [1]. Accumulation of Aβ in the brain is believed to result in formation of neurofibrillary tangles, inflammation, axonal injury, synapse loss, and neuronal apoptosis, leading to AD [2]. Thus, reducing Aβ levels should exert a neuroprotective effect against AD. Recent studies have shown that insulin-degrading enzyme (IDE) can effectively degrade Aβ in the brain [3], [4]. IDE, a highly conserved Zn(2+)-dependent endopeptidase, is known to degrade insulin and regulate the steady-state level of peripheral insulin. In addition, 3–10-kDa short peptides, including Aβ, are also substrates of IDE [5], [6], [7], [8].

Ginseng root has been used for several thousand years as a highly valued herb to treat weakness and fatigue, especially in China. The major active components of ginseng are ginsenosides, a diverse group of steroidal saponins, which target myriad tissues, producing an array of pharmacological responses [9]. Ginsenosides include Rb1, Rb2, Rc, Rd, Re, Rg1, and Rg2, with Rg1 being one of the most studied components. Rg1 exerts a neuroprotective effect and is beneficial in AD models *in vivo* and *in vitro* [10], [11].

Rg1, used as a small-molecule drug, can improve learning and memory in animals [12], [13], inhibit apoptosis induced by Aβ [14], alleviate oxidative stress [15], inhibit beta-secretase activity [11], maintain neuron activity at a normal level in hippocampus of a mouse model of Aβ-induced dementia [16], and improve neural plasticity [17]. Additionally, Rg1 has been recently used to treat type 2 diabetes, as it can improve peroxisome proliferator-activated receptor γ (PPARγ) expression and lipid metabolism [18].

PPARγ regulates IDE expression by binding to a peroxisome proliferator-response element (PPRE) in the IDE promoter [19]. As mentioned above, IDE participates in the proteolysis of Aβ [3], [4]. Therefore, we hypothesize that Rg1 increases IDE expression by upregulating PPARγ expression, and as a result, can decrease Aβ levels in the brain. To evaluate this hypothesis, we investigated the effects of Rg1 on learning and memory and hippocampal histopathological damage in a rat model of AD, while analyzing Aβ_1–42_, PPARγ, and IDE expression in the hippocampus. The results indicate that Rg1 can increase IDE expression by upregulating PPARγ, leading to decreased Aβ levels in the hippocampus, attenuated hippocampal histopathological abnormalities and improved learning and memory in a rat model of AD.

## Materials and Methods

### Ethics statement

All experimental protocols were approved by the Ethics Committee of the School of Life Science and Technology of Xi'an Jiaotong University.

### Materials

**Figure 1:**
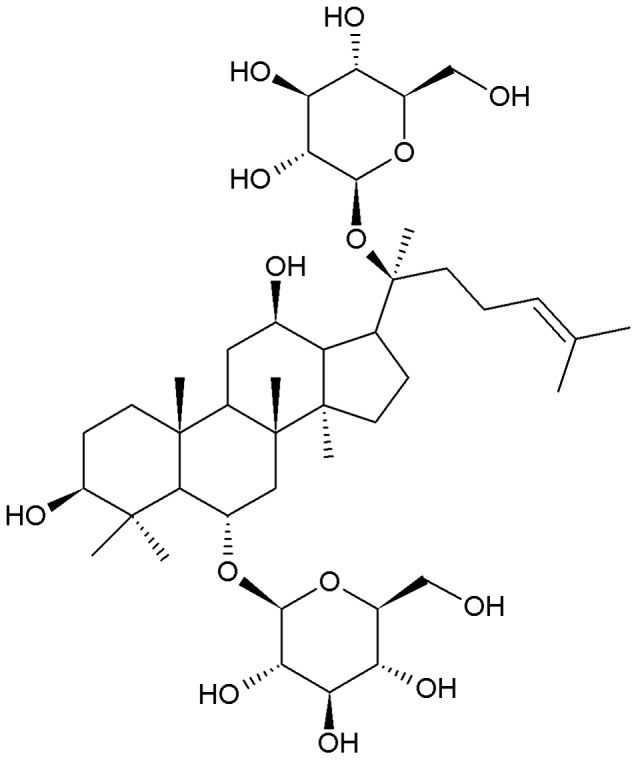
Chemical structure of ginsenoside Rg1.

Ginsenoside Rg1 was purchased from Hongjiu Biotech. Co., Ltd. (Jilin, China) in the form of white powder-like crystals, with a molecular weight of 801.01, general formula C_42_H_72_O_14_ (Figure 1), and a purity of over 98% as determined by HPLC. Rat Aβ_1–42_ and GW9662 were purchased from Sigma-Aldrich (St. Louis, MO, USA). Polyclonal rabbit anti-rat Aβ_1–42_, PPARγ and IDE antibodies were purchased from Abcam (Cambridge, UK). SP immunohistochemistry kit and DAB staining kit were purchased from Bioss (Beijing, China). Total protein extraction reagents and BCA protein assay kit were purchased from BestBio (Shanghai, China). PrimeScript™ RT reagent Kit (Perfect Real Time) and SYBR^®^ *Premix Ex Taq*™ II (Perfect Real Time) were purchased from TaKaRa (Shiga, Japan).

### Animals

Healthy male Sprague–Dawley rats (age 6–7 wks, weight 220±10 g) were purchased from the Experimental Animal Center of Xi'an Jiaotong University College of Medicine (License number: SCXK (Shaan) 2007-001). Rats were randomly divided into control, untreated, Rg1+GW9662, and Rg1 groups, with 10 animals in each group. Animals were housed in a room maintained at 23°C with a 12-hour light-dark cycle, and were allowed free access to food and water.

### Animal preparation

The rat model of AD was prepared as described previously [20], [21]. Aβ_1–42_ was diluted in sterile normal saline to a final concentration of 2 µg·µl^−1^. Rats were anesthetized with chloral hydrate (0.35 mg·kg^−1^, intraperitoneally) and fixed on a rat brain stereotaxic instrument. The scalp was incised, and the bregma and biparietal suture were exposed. Both hippocampal CA1 regions were chosen for injection of Aβ. Sites were verified in advance by injection of methylene blue solution. Holes were drilled in the skull using a dental drill, 2.2 mm from the biparietal suture and 3.0 mm behind the bregma. A microsyringe was then advanced 2.8 mm under the dura mater for injection of both hippocampal CA1 regions. Rats in untreated, Rg1+GW9662, and Rg1 groups were injected with 10 µg soluble Aβ_1–42_ at a rate of 0.5 µl·min^−1^, whereas the control group received sterile normal saline. The syringe was removed 5 min after injection. After surgery, the scalp was sutured, and sulfamethoxazole was sprinkled on the wound to prevent infection. In addition, penicillin (40,000 U) was injected intramuscularly into the gluteus, once a day for 3 days.

### Treatment

Drug treatment began after completion of the initial Morris water maze test. Rats in the Rg1 group were treated with Rg1 (10 mg·kg^−1^, intraperitoneally) [22] and rats in the Rg1+GW9662 group were treated with Rg1 (10 mg·kg^−1^, intraperitoneally) and GW9662 (1 mg·kg^−1^, intraperitoneally) [23], whereas animals in control and untreated groups were treated with the same volume of normal saline. Treatment occurred once a day for 28 days.

### Morris water maze test

The Morris water maze task was performed as described previously [24], [25], after Aβ injection and again after treatment. The maze was a tank (80 cm in radius and 45 cm high) filled with water at approximately 24°C. The tank was divided into 4 quadrants, one of which contained a circular escape platform (8 cm in diameter) placed at a fixed position, 2.5 cm below the surface of the water. There were visual cues around the water maze. Oriented navigation trials were performed 4 times per day, for 4 days. In each trial, the animal was placed into the water in a different quadrant and given 120 sec to search for the platform. If the rat escaped successfully onto the platform within the given time, it was allowed to stay on the platform for 10 sec. If the rat failed to find the platform within the given time, it was guided to the platform and allowed to stay for 10 sec, after which time, a new trial would begin. Behavior was recorded by a computerized video tracking system, and the time that a rat took to reach the submerged platform (escape latency) was recorded to assess spatial learning ability. On the fifth day, the platform was removed from the tank, and a spatial probe trial was performed. Rats were placed in the tank in the quadrant opposite to the quadrant that previously held the platform, and were allowed to swim freely for 120 sec. The average search time rats spent in the target quadrant was recorded to assess spatial memory ability. The target quadrant was defined as the quadrant that previously held the platform, whose radius was limited to 70 cm in this assessment.

### Tissue preparation

After water maze testing was completed, 3 rats in each group were selected randomly for immunohistochemistry staining. These rats were anesthetized with chloral hydrate and the heart was exposed. Cold normal saline was perfused into the aorta through a left ventricular catheter for 1 min; subsequently, 4% paraformaldehyde was perfused until the tail and limbs were rigid. The brain was removed and cut coronally into 3 portions, at sites 2 mm and 4 mm behind the bregma, and the middle portion was postfixed in 4% paraformaldehyde and embedded in paraffin. Sections (4 µm) were cut coronally at a site 3 mm behind the bregma for hematoxylin-eosin (HE) staining and immunohistochemical analysis. The remaining rats in each group were decapitated after anesthesia without perfusion, and the hippocampus was dissected and stored in liquid nitrogen for analysis by western blotting and real-time PCR.

### HE staining

In brief, after the paraffin sections were dewaxed, hematoxylin staining was performed for 3 min, followed by eosin staining for 3 s, and then the sections were dehydrated with alcohol, made hyaline with xylene, and sealed. The hippocampal histopathological abnormalities were investigated under a light microscope. The number of cells in the hippocampal CA1 region of each section was examined by 2 pathologists in a blinded manner, and the average number was taken as the final result.

### Immunohistochemistry

Immunohistochemical staining was performed using the SP immunohistochemistry kit according to the manufacturer's instructions using a method described previously [26]. Sections were dewaxed, and then subjected to heat-mediated antigen retrieval with 0.01 M citric acid buffer (pH 6.0). Following several washes in PBS, sections were blocked with 10% goat serum, and then incubated overnight with polyclonal rabbit anti-rat Aβ_1–42_ antibody (1∶600), PPARγ antibody (1∶400), and IDE antibody (1∶1300) at 4°C overnight. PBS was used in place of primary antibody as a negative control. After incubation with secondary antibody, immunoreactivity was detected with a DAB staining kit, and sections were counterstained with hematoxylin.

### Western blot

The right hippocampus, stored in liquid nitrogen, was homogenized and total proteins were extracted using the total protein extraction reagents kit. Protein concentration was measured using the BCA protein assay kit. After denaturation at 95°C, proteins were separated by electrophoresis. Separated proteins were transferred onto nitrocellulose membranes, which were washed in TBST before incubation in 5% skim milk (diluted in TBST) at 37°C for 1 h. Blots were incubated overnight with polyclonal rabbit anti-rat Aβ_1–42_ antibody (1∶500), PPARγ antibody (1∶600), and IDE antibody (1∶600) at 37°C, and then incubated with secondary antibody at 37°C for 1 h. Immunoreactive bands were detected by enhanced chemiluminescence. Bands were analyzed using ImageJ software (version 1.44p, USA). Values obtained were normalized basing on density values of internal β-actin.

### Real-time PCR

In this study, mRNA level for Aβ_1–42_ was not analyzed because Aβ_1–42_ in the hippocampus was injected exogenously. Total RNA was extracted from the left hippocampus, stored in liquid nitrogen, using Trizol reagent, and RNA concentration was determined using UV spectrophotometry. cDNA was synthesized by reverse transcription using PrimeScript™ RT reagent Kit (Perfect Real Time). Primers were designed by Primer Premier 5.0 according the mRNA sequences of PPARγ and IDE genes retrieved from GenBank, and synthesized by Sangon Biotech (Shanghai) Co., Ltd. in China. Primer sequences were as follows: PPARγ forward primer 5′ GATGACCACTCCCATTCCTTT3′, reverse primer 5′ CGCACTTTGGTATTCTTGGAG3′, 156 bp; IDE forward primer 5′ TCTGAGCCTTGCTTCAACACT3′, reverse primer 5′ TGAGGTGGTTTTTCTGACTGG3′, 125 bp; β-actin forward primer 5′ CCCATCTATGAGGGTTACGC3′, reverse primer 5′ TTTAATGTCACGCACGATTTC3′, 150 bp. Real-time PCR was performed on the ABI 7500 PCR system using SYBR^®^ *Premix Ex Taq*™ II (Perfect Real Time) according to the manufacturer's instructions. PCR conditions were as follows: 94°C for 4 min; then 30 cycles of 94°C for 45 s, 61°C for 60 s, and 72°C for 90 s. Cycle threshold values were obtained from the ABI 7500 sequence detection system software. Data were analyzed using the ΔΔCt method and β-actin served as the internal control.

### Statistical analysis

Data are presented as mean±SEM and were analyzed by the SPSS13.0 software. Two-way repeated measures analysis of variance and post-hoc least significant difference (LSD) test were used to analyze latency in the Morris water maze test. One-way analysis of variance and post-hoc LSD test were used to analyze time in target quadrant in the Morris water maze test and the expression of Aβ_1–42_, IDE, and PPARγ in different groups. A *P* value<0.05 denoted a significant statistical difference.

## Results

### Morris water maze test

**Figure 2:**
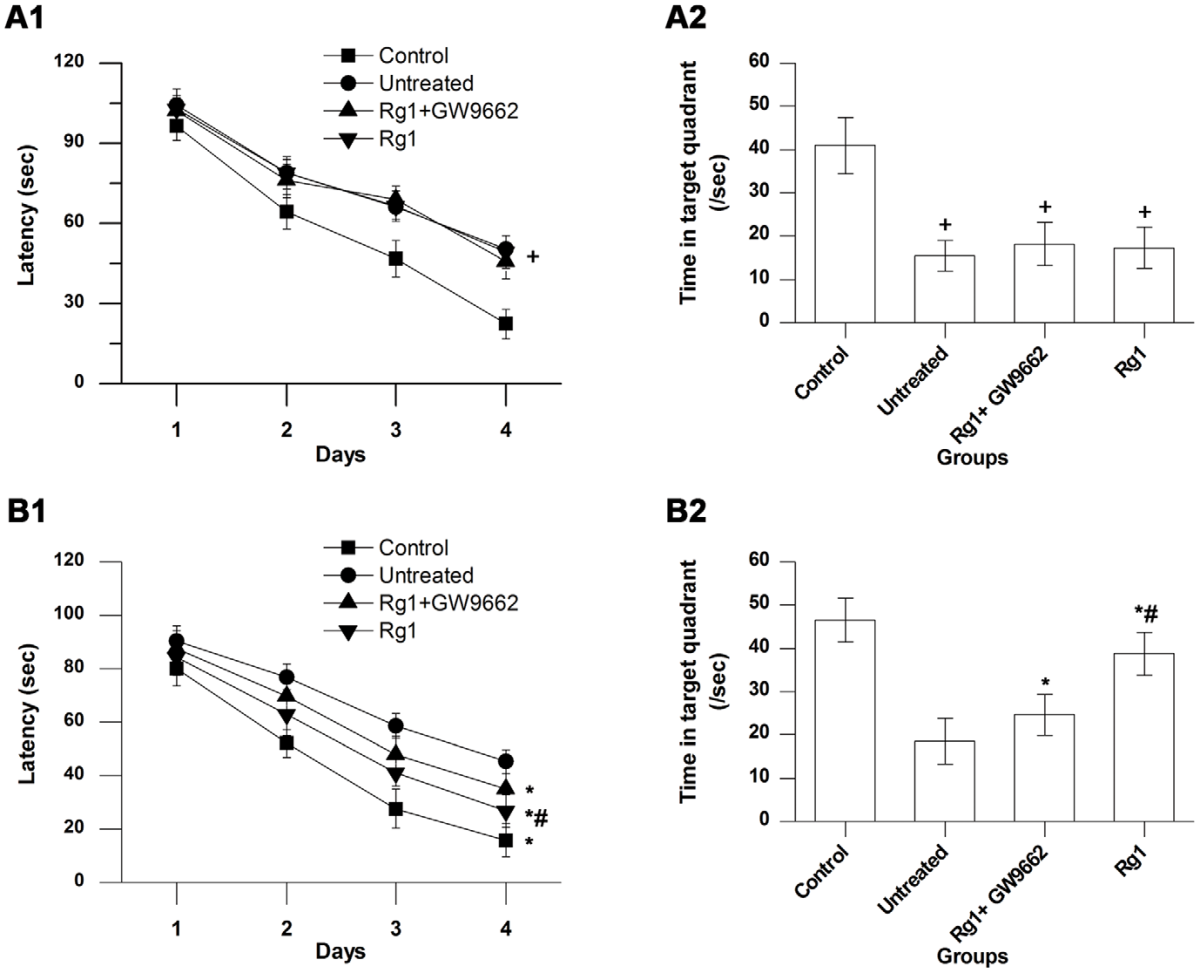
Results of the Morris water maze test. **A**, comparisons of average latency in the oriented navigation trial (A1) and average time spent in the target quadrant in the probe trial (A2) after Aβ_1–42_ injection. **B**, comparisons of average latency (B1) and average time spent in the target quadrant (B2) after Rg1 treatment. Control group, intrahippocampal injection of normal saline and intraperitoneal injection of normal saline; Untreated group, intrahippocampal injection of Aβ_1–42_ and intraperitoneal injection of normal saline; Rg1+GW9662 group, intrahippocampal injection of Aβ_1–42_ and intraperitoneal injection of Rg1 and GW9662; Rg1 group, intrahippocampal injection of Aβ_1–42_ and intraperitoneal injection of Rg1. Bars represent mean±SEM. *n* = 10. +,vs. control group, *P*<0.05. *, vs. untreated group, *P*<0.05. #, vs. Rg1+GW9662 group, *P*<0.05.

The effect of Aβ_1–42_ injection on cognitive function in rats was assessed by the Morris water maze test. The results are shown in Figure 2A. There was no significant difference in swim speed among the groups (not shown). Animals in the untreated, Rg1+GW9662, and Rg1 groups performed poorly, exhibited longer latency on the oriented navigation trial (Figure 2A1), and spent less time in the target quadrant in the spatial probe trial (Figure 2A2) than the rats in control group (*P*<0.05 for all), whereas there was no significant difference among the 3 groups (*P*>0.05). The water maze test was performed again to determine the effect of Rg1 treatment on cognitive function. The results are shown in Figure 2B. Compared with the untreated group, animals in the Rg1 exhibited shorter latency (*P*<0.05) (Figure 2B1) and more time in the target quadrant (*P*<0.05) (Figure 2B2). Compared with the Rg1 group, animals in the Rg1+GW9662 group showed longer latency (*P*<0.05) and less time in the target quadrant (*P*<0.05). The result indicates that Rg1 treatment can improve spatial learning and memory in this rat model of AD. Moreover, the results show that the effect of Rg1 treatment could be blocked effectively by GW9662, a PPARγ antagonist.

### HE staining

**Figure 3:**
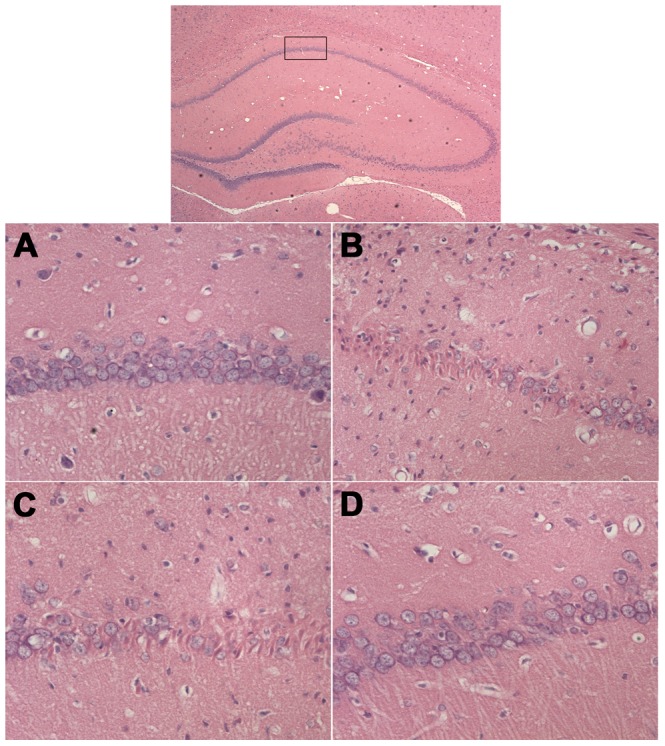
HE staining (×400). **A**, control group; **B**, untreated group; **C**, Rg1+GW9662 group; **D**, Rg1 group. Control group, intrahippocampal injection of normal saline and intraperitoneal injection of normal saline; Untreated group, intrahippocampal injection of Aβ_1–42_ and intraperitoneal injection of normal saline; Rg1+GW9662 group, intrahippocampal injection of Aβ_1–42_ and intraperitoneal injection of Rg1 and GW9662; Rg1 group, intrahippocampal injection of Aβ_1–42_ and intraperitoneal injection of Rg1. Rats in the control group did not show histopathological abnormalities. In the untreated and Rg1+GW9662 groups, the number of cells in the hippocampal CA1 region appeared decreased. Furthermore, the remnants of the pyramidal cells were arranged irregularly and some exhibited shrunken and irregular shape. The cells in the Rg1 group had better cell morphology and were more numerous than those in the untreated and Rg1+GW9662 groups.

HE staining revealed no remarkable neuronal abnormalities in the hippocampus of rats in the control group. The pyramidal cells in the CA1 region were arranged neatly and tightly, and no cell loss was found. Additionally, for the control group, cells were round and intact with nuclei stained clear, dark blue (Figure 3A). However, obvious hippocampal histopathological damage was observed in the untreated and Rg1+GW9662 groups. The pyramidal layered structure was disintegrated, and neuronal loss was found in the CA1 region. Neurons with pyknotic nuclei and with shrunken or irregular shape were also observed (Figure 3B and 3C). These abnormalities were attenuated by Rg1 treatment. The cells in Rg1 group had better cell morphology and were more numerous than those in the untreated and Rg1+GW9662 groups, but were overall worse than those in the control group (Figure 3D). The average number of cells was highest in the control (67) and Rg1 group (59), lower in the Rg1+GW9662 group (51), and lowest in the untreated group (48).

### Immunohistochemistry

**Figure 4:**
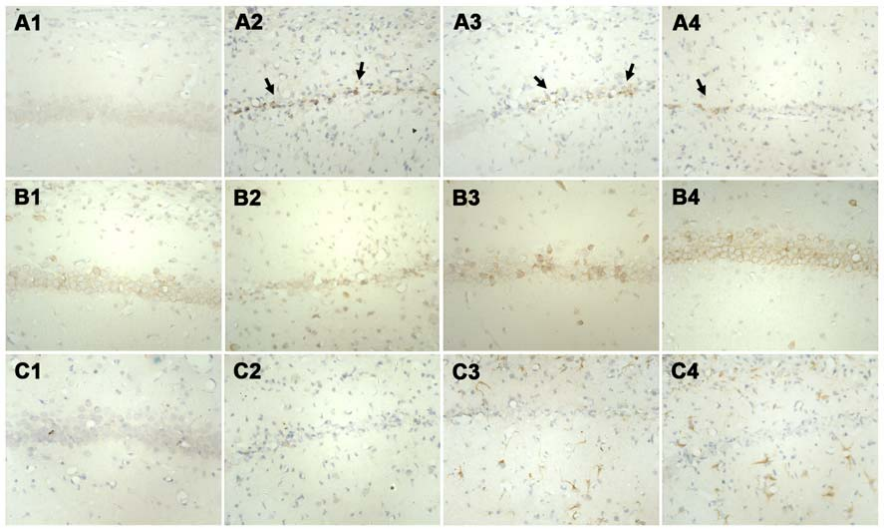
Immunohistochemical staining of the CA1 region of the hippocampus (×400). **A**, **B**, and **C**, staining for Aβ_1–42_, IDE, and PPARγ, respectively. 1, 2, 3, and 4: control, untreated, Rg1+GW9662, and Rg1 groups, respectively. Control group, intrahippocampal injection of normal saline and intraperitoneal injection of normal saline; Untreated group, intrahippocampal injection of Aβ_1–42_ and intraperitoneal injection of normal saline; Rg1+GW9662 group, intrahippocampal injection of Aβ_1–42_ and intraperitoneal injection of Rg1 and GW9662; Rg1 group, intrahippocampal injection of Aβ_1–42_ and intraperitoneal injection of Rg1. Arrows indicate exogenous Aβ_1–42_.

We investigated the distribution of Aβ_1–42_, PPARγ, and IDE in the hippocampus and the effect of Rg1 by immunohistochemical staining. We found some extracellular exogenous Aβ_1–42_ in the hippocampus of Aβ_1–42_-injected rats, as indicated by tan color (arrows in Figure 4A); furthermore, some intracellular Aβ_1–42_ was also detected. Exogenous Aβ_1–42_ appeared highest in the untreated group, less in the Rg1+GW9662 group, and lowest in the Rg1 group, and no extracellular exogenous Aβ_1–42_ was detected in the control rats. Additionally, PPARγ- and IDE-immunoreactive cells were most numerous in the Rg1 group, less in the Rg1+GW9662 group, and the least in the control and untreated groups (Figure 4B and 4C).

### Western blot

**Figure 5:**
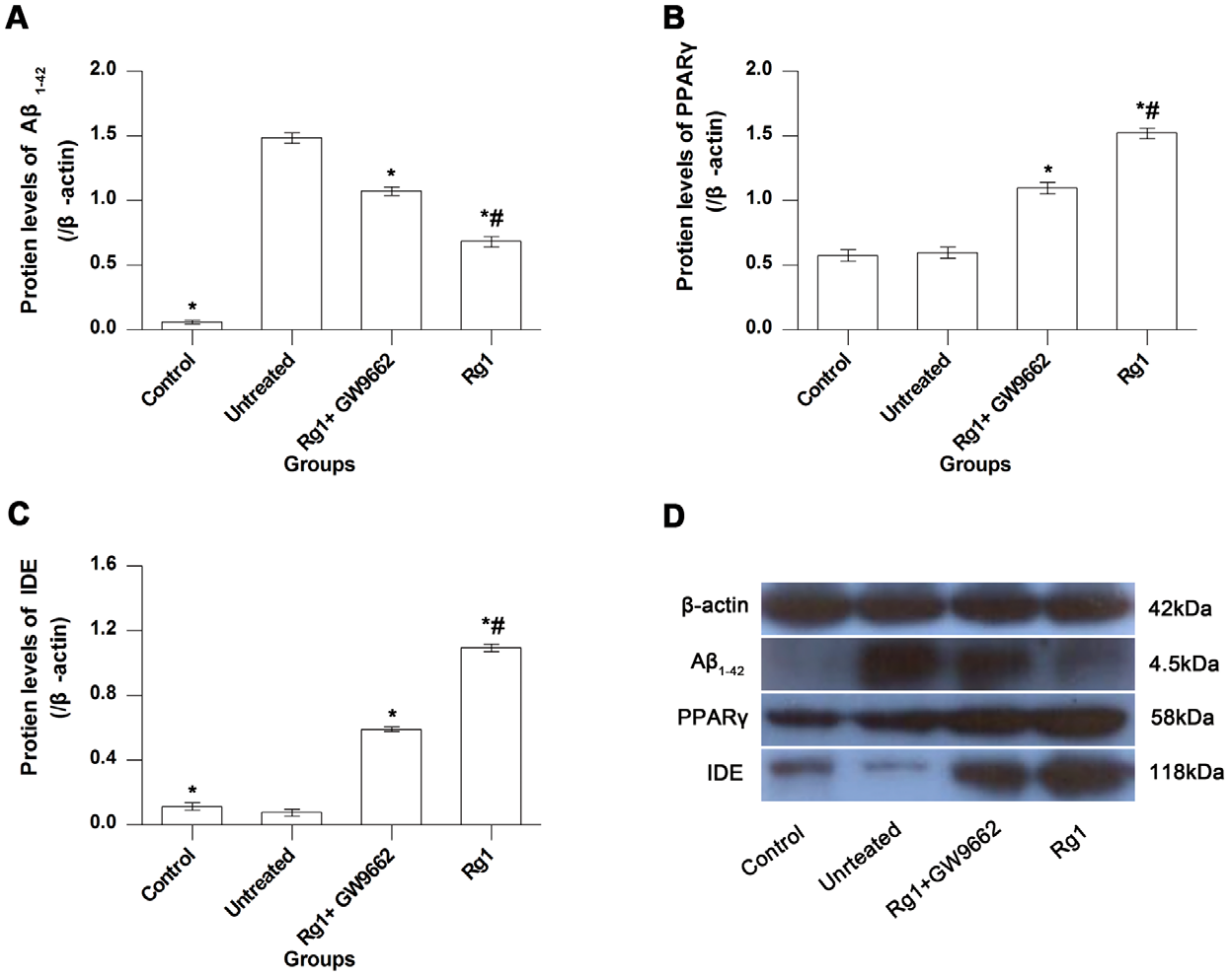
Western blot results. **A**, **B**, and **C**, comparisons of protein levels of Aβ_1–42_, PPARγ, and IDE among control, untreated, Rg1+GW9662, and Rg1 groups, respectively. **D**, detection of Aβ_1–42_, PPARγ, and IDE proteins by western blot. Control group, intrahippocampal injection of normal saline and intraperitoneal injection of normal saline; Untreated group, intrahippocampal injection of Aβ_1–42_ and intraperitoneal injection of normal saline; Rg1+GW9662 group, intrahippocampal injection of Aβ_1–42_ and intraperitoneal injection of Rg1 and GW9662; Rg1 group, intrahippocampal injection of Aβ_1–42_ and intraperitoneal injection of Rg1. Bars represent mean±SEM. *n* = 7. *, vs. untreated group, *P*<0.05. #, vs. Rg1+GW9662 group, *P*<0.05.

Western blot analysis for Aβ_1–42_ is shown in Figure 5A. Little Aβ_1–42_ was detected in control rats, but a large quantity of Aβ_1–42_ was detected in the hippocampus of Aβ_1–42_-injected animals (control group vs. untreated group, *P*<0.05). This is consistent with results of immunohistochemistry; taken together with the water maze test results, these data indicate that the rat model of AD prepared by injecting soluble Aβ_1–42_ into CA1 regions is valid.

Aβ_1–42_ protein levels in the hippocampus of the Rg1 group were lower than those in the hippocampus of the untreated group (*P*<0.05). Thus, Rg1 treatment effectively reduced the protein level of Aβ_1–42_ in the hippocampus; this effect could be blocked by GW9662 treatment (Rg1 group vs. Rg1+GW9662 group, *P*<0.05), although the inhibition was incomplete (untreated group vs. Rg1+GW9662 group, *P*<0.05). For PPARγ protein, there was no significant difference between control and untreated groups (*P*>0.05), but the protein level in the Rg1 group was higher than in the untreated group (*P*<0.05) (Figure 5B). Thus, Rg1 effectively upregulated PPARγ expression in the hippocampus, and this effect could be inhibited incompletely by GW9662 (Rg1 group vs. Rg1+GW9662 group, *P*<0.05). IDE expression is shown in Figure 5C. Compared with the control group, the protein level in the untreated group was lower (*P*<0.05). Compared with the untreated group, the IDE protein level was higher in the Rg1 and Rg1+GW9662 groups (*P*<0.05 in both). Hence, Rg1 upregulated IDE expression in the hippocampus, and this effect could be inhibited incompletely by GW9662.

### Real-time PCR

**Figure 6:**
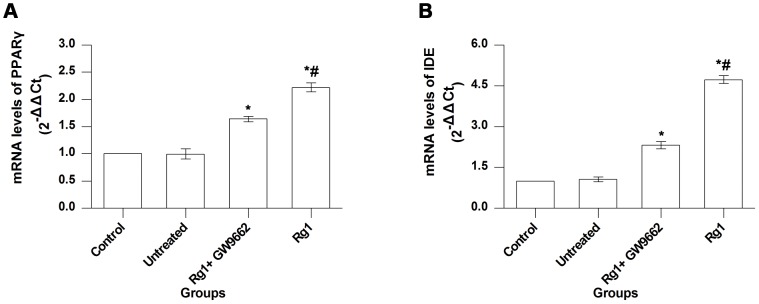
Real-time PCR results. **A**, **B**, comparisons of mRNA levels of PPARγ and IDE among control, untreated, Rg1+GW9662, and Rg1 groups. Control group, intrahippocampal injection of normal saline and intraperitoneal injection of normal saline; Untreated group, intrahippocampal injection of Aβ_1–42_ and intraperitoneal injection of normal saline; Rg1+GW9662 group, intrahippocampal injection of Aβ_1–42_ and intraperitoneal injection of Rg1 and GW9662; Rg1 group, intrahippocampal injection of Aβ_1–42_ and intraperitoneal injection of Rg1. Bars represent mean±SEM. *n* = 7. *, vs. untreated group, *P*<0.05. #, vs. Rg1+GW9662 group, *P*<0.05.

PPARγ mRNA levels were higher in the Rg1 group than in the control, untreated, and Rg1+GW9662 groups (*P*<0.05 for all). Expression in the Rg1+GW9662 group was higher than that in the control and untreated groups (*P*<0.05 for both), and there was no significant difference between the control and untreated groups (*P*>0.05) (Figure 6A). IDE mRNA expression level was the highest in the Rg1 group (Rg1 group vs. other groups, *P*<0.05), lower in the Rg1+GW9662 group (Rg1+GW9662 group vs. control and untreated groups, *P*<0.05 for both), and was the lowest in control and untreated groups (Figure 6B). There was no significant difference between control and untreated groups (*P*>0.05).

## Discussion

Recently, some PPARγ agonists such as thiazolidinediones (TZDs), have been tested as treatments for AD in vivo and in vitro [27], and some progress has been made. For example, diet-induced insulin resistance in rats could induce Aβ overproduction and reduced IDE activity, and pioglitazone treatment could prevent these abnormalities [1]. Rosiglitazone reduced Aβ level and rescued memory impairment in Alzheimer's transgenic mice [28]. There are 2 reasons for using PPARγ agonists to treat AD, as previous studies have shown. First, insulin resistance (IR) is closely related to the pathogenesis of AD. Previous studies have shown that hyperinsulinemia induced by IR can increase the incidence of AD [29], [30]. Indeed, AD is referred to as type 3 diabetes by some scholars [31], [32]. As both insulin and Aβ are IDE substrates, insulin may compete with Aβ for access to IDE [33], [34]. In IR, insulin levels are abnormally high, increasing the amount of insulin competing with Aβ for IDE in the brain [2], [33], [35]. The result is that Aβ is degraded less effectively and the level of Aβ in the brain increases. This excessive Aβ could aggregate and then deposit in the brain, ultimately leading to the pathological changes characteristic of AD, including inflammation, formation of senile plaques, formation of neurofibrillary tangles, and neuronal apoptosis [2]. PPARγ agonists can increase insulin sensitivity and downregulate insulin levels, and consequently inhibit insulin competing with Aβ for IDE.

Second, PPARγ can induce IDE expression at the transcriptional level [19]. Therefore, we can infer that PPARγ agonists upregulate IDE expression, leading to enhanced degradation of Aβ. In fact, this effect of PPARγ agonists has been demonstrated in several studies [1], [28], though the mechanism is still unknown.

At present, thiazolidinediones (TZDs) are extensively used as PPARγ agonists, and their use is a new strategy for AD treatment [1], [28]. However, the pathogenesis of AD is complicated, and long-term medication is necessary for AD treatment. TZDs are associated with several side effects, including fractures [36], heart failure [37], [38], stroke [38], and even bladder cancer [39], suggesting that these drugs are probably not fit for AD treatment. Thus, it is important to find an alternative drug that can increase IDE expression by activating or upregulating PPARγ with minimal side effects, and thereby be recommended as safe and effective for AD treatment.

As one of the main active ingredients in ginseng, Rg1 is generally regarded to be beneficial for neurodegenerative diseases, with few side effects. Recently, it has been suggested that Rg1 is also useful for treatment of type 2 diabetes. Specifically, Rb1 and Rg1 treatment improved PPARγ expression and decreased total cholesterol, triglyceride, and glucose levels in peripheral blood of patients with type 2 diabetes [18]. However, it is not clear whether Rg1 or Rb1 played the key role in improving PPARγ expression.

Here, we show that Rg1 could improve PPARγ expression in the hippocampus in a rat model of AD. The results establish that Rg1 is an acceptable substitute for TZDs in research on AD therapy because of its ability to upregulate PPARγ expression. We further investigated expression levels of Aβ and IDE in the hippocampus, and used immunohistochemistry, western blotting, and real-time PCR, to show that expression of PPARγ and IDE was increased and expression of Aβ was decreased in the hippocampus after Rg1 treatment. This effect could be reversed effectively by GW9662, a PPARγ antagonist. Consistent with our hypothesis, Rg1 could increase IDE expression by upregulating PPARγ expression, leading to decreased Aβ level in the brain, and as a result, attenuated hippocampal histopathological abnormalities and improved spatial learning and memory in a rat model of AD. However, it is not possible to determine whether Rg1 acts solely as a PPARγ agonist by observing the upregulation of PPARγ and IDE.

Additionally, there is an interesting and unexplained anomaly in the present study. The IDE level in the hippocampus in Aβ_1–42_-injected rats was lower than that in the control rats. It is difficult to explain this observation. In fact, the correlation between IDE and Aβ remains controversial and uncertain. Some reports showed that IDE protein concentrations and activity are decreased in the brains of AD patients [40], [41] and that IDE protein activity is negatively correlated with brain Aβ_1–42_ content [40]. However, an apparently opposite conclusion was reached in some animal experiments. Vepsalainen et al. showed that mRNA and protein levels of IDE were significantly upregulated in brains of transgenic mice and that upregulation of IDE mRNA levels occurred in parallel with increased Aβ_1–40_ and Aβ_1–42_ production [42]. Therefore, it is extremely important to clarify the correlation between IDE and Aβ in future studies.

We also found that GW9662 could not completely block the effect of Rg1 on Aβ levels. This suggests that Rg1 affects Aβ degradation through other mechanisms. Aβ is degraded by many proteases, including neprilysin [43], [44], endothelin-converting enzyme [45], [46], angiotensin-converting enzyme [47], plasminogen activator [48], [49], and matrix metalloproteinase-9 [50]. In addition, Aβ can be transported across the blood-brain barrier [51], [52]. However, whether these mechanisms explain the clearing effect of Rg1 on Aβ is not clear. Of course, partial inhibition by GW9662 may result from a submaximal dose of GW9662 in the experiment. In addition, GW9662 did not completely block the effect of Rg1 on PPARγ and IDE expression, indicating that that Rg1 affects PPARγ and IDE expression through additional pathways. Further research is needed to clarify these points.

## Conclusions

In summary, our data show that Rg1 increased IDE expression by upregulating PPARγ, leading to decreased Aβ_1–42_ levels in the hippocampus, and as a result, attenuated hippocampal histopathological abnormalities and improved spatial learning and memory in a rat model of AD. These findings suggest that Rg1 is a promising new therapeutic agent for the treatment of AD.
